# Estimating Iowa’s riverine phosphorus concentrations via water quality surrogacy

**DOI:** 10.1016/j.heliyon.2024.e37377

**Published:** 2024-09-03

**Authors:** Elliot S. Anderson, Keith E. Schilling, Chris S. Jones, Larry J. Weber

**Affiliations:** a Iowa Geological Survey, University of Iowa, Iowa City, IA, USA; b IIHR-Hydroscience & Engineering (retired), University of Iowa, Iowa City, IA, USA; c IIHR-Hydroscience & Engineering, University of Iowa, Iowa City, IA, USA

**Keywords:** Phosphorus, Turbidity, Surrogacy, Iowa

## Abstract

Phosphorus (P) is a widespread waterborne pollutant that impairs many waterbodies. However, it is challenging to measure directly, and much research has been dedicated to developing surrogacy models that can repeatedly predict its concentration. Optimal approaches for modeling strategies are often unclear and depend upon local P dynamics and the availability of financial and technical resources. This study presents a schema for developing P surrogacy models at a statewide scale (16 major rivers in Iowa, USA). Specifically, we examined the relationship between particulate phosphorus (Part P) and orthophosphate (OP) and explored the viability of eight potential surrogates in predicting their concentrations using multiple linear regression and power regression methods. We also investigated similarities between surrogate models for Part P and total suspended solids (TSS). At all sites, OP and Part P were not strongly correlated (mean R = 0.20 ± 0.17). Many instances were observed where samples had high concentrations of one form but not the other. Modeling results demonstrated that turbidity was consistently the best predictor (t-statistics >10) of Part P, and adding other surrogates alongside turbidity did little to improve model performance. No surrogates proved useful in estimating OP. Viable power regression models were created using turbidity to predict Part P (mean R^2^ = 0.69 ± 0.12). These models had a nonlinear form where Part P concentrations leveled off as waters became exceptionally turbid. This contrasted with TSS, which maintained a strong linear relationship across all turbidity levels. Turbidity-based models show promise in quantifying statewide P levels, as they enable high-resolution and real-time Part P estimates.

## 1 Introduction

Phosphorus (P) is an essential nutrient needed by all plants and animals to survive [1,2] but can become an environmental pollutant when it enters surface water [3]. Impairments driven by P pollution are some of the most pressing challenges facing water quality in the State of Iowa [4,5] and the greater U.S. Midwest [6,7]. Stakeholders are working to devise solutions that would reduce P levels in Iowa’s lakes and streams [8]. This effort was formalized as part of the Iowa Nutrient Reduction Strategy (INRS), a plan that identifies actions needed to reduce P loads by 45 % in Iowa rivers [9]. The INRS was formed to align with the broader Gulf of Mexico Hypoxia Action Plan, which aims to comprehensively lessen all nutrients transported to the Gulf of Mexico [10]. The INRS designated best practices to prevent P from entering Iowa’s waters by inhibiting its transport through various pathways [11].

Evaluating the effectiveness of these strategies is not always straightforward [12]. It requires accurate measurements of P concentrations and loads, but it is impractical to measure P in every waterbody in a state with >100 lakes and >100,000 km of perennial streams. The State of Iowa focused on quantifying the total amount of P exported from the state rivers as a key metric for gauging the progress of the state’s remediation efforts. However, there are several challenges in estimating how much P is exported by Iowa’s rivers [13]. First, P is difficult and expensive to measure directly [14]. Samples must be collected onsite, brought to a lab, and carefully measured through several analytical chemistry procedures [15]. Second, P concentrations in rivers and streams can change rapidly over short periods of time [16]. These rapid fluctuations mean that it is difficult to quantify P during non-sampling periods. Current and historical P sampling of Iowa’s principal waterbodies has been monthly—not nearly frequent enough to fully evaluate its presence [13].

Furthermore, waterborne P exists in multiple chemical forms, each with different sources [17] and environmental pathways [18]. Total phosphorus (TP), a measure of all aquatic forms of P, consists of dissolved and particulate components. In Iowa, orthophosphate (OP) makes up the vast majority of dissolved P [19]. Although there are commercially-available devices that measure OP in-situ, the technology has not developed well enough to enable large-scale deployments [20]. Particulate phosphorus (Part P) contains all the suspended P forms present in a water column. Part P is often sorbed directly to soil particles [21] but can also exist as organic P in water or as any mineral that may precipitate out of the water solution [22]. Part P cannot be measured directly [23]. Instead, it is inferred within a sample by subtracting dissolved P from TP.

Streamflow records are also needed to estimate a river’s TP load for the desired period. Streamflow multiplied by the TP concentration yields the P load for an increment of time. In Iowa and throughout the U.S., streamflow measurements are routinely made by the United States Geological Survey (USGS) at stream gauge stations placed along rivers, with 15-min and daily average flows and stage data available for 124 locations in Iowa.

Because P concentrations are dynamic in river systems, it is important that sampling strategies or statistical models account for temporal variability. A common strategy for estimating riverine P is the use of water quality surrogates [24,25]. Surrogates are water-related parameters that can be measured reliably onsite over continuous periods [26,27]. By definition, surrogates infer information about one parameter from indirect data related to another parameter [28,29]. Most surrogacy models have used straightforward regression relationships to describe this inference [30]. Common surrogates include streamflow, temperature, specific conductivity (SC), and turbidity [31]. While surrogates can be measured at a far more frequent temporal scale than P, they rely on consistent in-situ observations and are thus still subject to practical considerations involving equipment cost and maintenance and issues surrounding sensor fouling.

In several studies, turbidity has been identified as a capable surrogate for TP [12,20,30,[32], [33], [34]]. Turbidity is a quantitative measure of water’s clarity. Turbidimeters measure this value by tracking how much light is scattered by particles within the water—a process known as nephelometry [35,36]. By deploying a turbidimeter in a river, continuous turbidity measurements can be collected at very high resolutions [37,38], usually reported as nephelometric turbidity units (NTU).

Most surrogacy models that predict P have traditionally used linear or power regression relationships to link turbidity (as the independent variable) to TP (as the dependent variable) [39]. For each location, the parameters for each equation are estimated. Each regression equation is considered to be site-specific (the parameters are not interchangeable between sites [40]), since soil characters, topography, and upstream land use conditions often cause variations in the regression models between locations [20]. Site specificity is critical, and a new model should be developed for each site of interest [41]. While model results have demonstrated considerable variability, this general strategy has proven effective across a wide range of climates, watershed characteristics, and hydrologic conditions [12,32,34,37,39,[42], [43], [44]].

More sophisticated modeling techniques have also been developed for water quality surrogates [[45], [46], [47]], especially with advancements in machine learning methods [48,49]. Many models have proven effective at predicting P concentrations [26,50,51], but these models often lack interpretability [26,51], which is essential in understanding P’s transport mechanisms [52] and pathways [53] and ultimately devising strategies for its remediation [54,55]. Most efforts interested in describing P at a regional scale (e.g., >HUC08) opt for a simpler model approach [20,56]. However, this sort of analysis, where the relationships between various forms of P and potential surrogates are systematically evaluated, has not been conducted comprehensively at a statewide scale.

The purpose of this study was to investigate the use of water quality surrogates to estimate P concentrations in Iowa’s rivers (i.e., a statewide scale). Our specific objectives were to: 1) identify Iowa locations for a surrogacy-based analysis and assemble relevant P and surrogate datasets; 2) explore the relationship between riverine P forms in Iowa, specifically Part P and OP; 3) evaluate the statistical significance between several potential surrogates and various forms of P; and 4) construct final models that can be implemented to predict P concentrations. Results from this study have both local and global implications. Locally, our models can be utilized to track the performance of statewide P reduction strategies to reduce P export from Iowa. Globally, our methods can be applied to explore potential surrogacy models in any locale plagued by P with adequate water quality data.

## 2 Methods

### 2.1 Site selection and data assembly

**Fig. 1:**
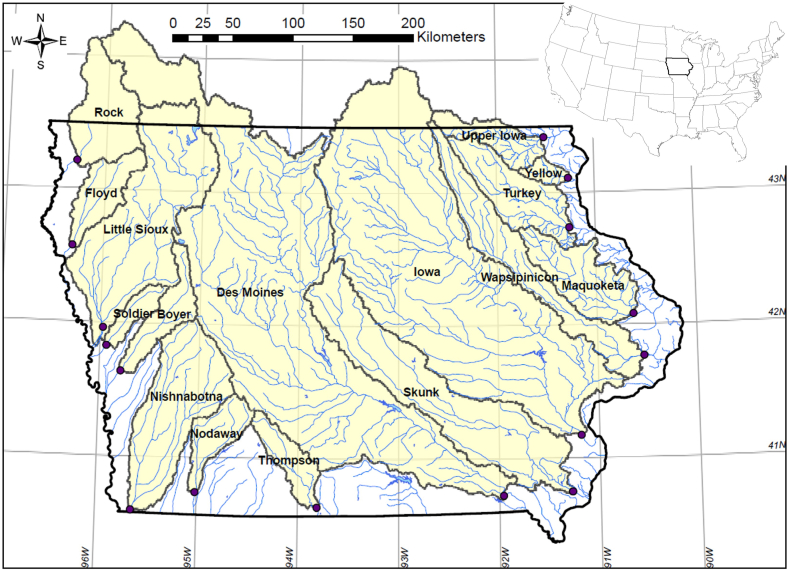
Iowa's 16 terminal monitoring sites and their corresponding watersheds.

Our analysis centered on 16 locations along Iowa’s major rivers—hereafter referred to as the terminal monitoring sites (Fig. 1). These locations lie near the state border and are the most downstream USGS stream gauges within Iowa for its major rivers. The total tributary areas cover approximately 90 % of the total land area in Iowa. The watersheds for these rivers span a variety of landform regions [57] and range from large watersheds containing flood control impoundments, such as the Des Moines and Iowa Rivers, to smaller, unimpounded watersheds, such as the Yellow and Soldier Rivers.

Long-term (>20 years), monthly water quality data have been collected at each site by the Iowa Department of Natural Resources (IDNR) as part of an ambient water monitoring program [58]. In addition, the USGS has also monitored water quality at several of the terminal sites. Nine rivers (Boyer, Des Moines, Iowa, Little Sioux, Maquoketa, Nishnabotna, Skunk, Turkey, and Wapsipinicon) were monitored twice a month from 2004 to 2014 [59]. Among the constituents measured by the IDNR and USGS are TP, OP, and several potential P surrogates.

Both datasets that were retrieved for this study are publicly available in the EPA STORET database. The datasets were merged to obtain robust records of P and corresponding potential surrogates, and sample counts ranged from 250 to 500 measurements per site. All water quality data were collected from surface waters following quality assurance protocols provided by the Environmental Protection Agency, and collection methods remained unchanged throughout the sampling timeframe. Subsamples were collected and filtered on-site, which were used to quantify dissolved constituents, such as OP.

Coincident streamflow was obtained for each measurement by pairing the water quality samples with daily mean flows observed at the co-located USGS stream gauges. Each gauge uses standard UGSG protocols to estimate flow (i.e., site-specific rating curves relating stage and discharge) every 15 min. Daily mean flow values are the arithmetic means of these 15-min estimates. Table 1 summarizes relevant information for each terminal monitoring site, including the IDNR and USGS site IDs. All metadata for these locations is publicly available on the IDNR and USGS web pages.

**Table 1:** Iowa’s 16 terminal monitoring sites and sample counts of their respective P data.

Short Name	Full Name	IDNRid	USGSid	TP samples	OP samples	Area (km^2^)	Lat	Long
Boyer	Boyer River at Logan, IA	10430001	06609500	397	399	2,256	41.64169	−95.7823
Des Moines	Des Moines River at Keosauqua, IA	10890001	05490500	509	510	36,358	40.72781	−91.9596
Floyd	Floyd River at James, IA	10750001	06600500	306	306	2,295	42.57666	−96.3114
Iowa	Iowa River at Wapello, IA	10580003	05465500	496	497	32,375	41.17809	−91.1821
Little Sioux	Little Sioux River near Turin, IA	10670003	06607500	254	254	9,132	41.96503	−95.9723
Maquoketa	Maquoketa River near Green Island, IA	10490005	05418500	254	254	4,022	42.08335	−90.6329
Nishnabotna	Nishnabotna River above Hamburg, IA	10360003	06810000	255	255	7,268	40.60167	−95.645
Nodaway	Nodaway River at Clarinda, IA	10730001	06817000	274	273	1,974	40.74328	−95.0142
Rock	Rock River near Rock Valley, IA	10840001	06483500	276	273	4,123	43.21443	−96.2945
Skunk	Skunk River at Augusta, IA	10560002	05474000	288	289	11,168	40.75365	−91.2771
Soldier	Soldier River at Pisgah, IA	10430002	06608500	284	284	1,054	41.83054	−95.9314
Thompson	Thompson River at Davis City, IA	10270001	06898000	280	278	1,816	40.64028	−93.8083
Turkey	Turkey River at Garber, IA	10220001	05412500	449	415	4,002	42.73999	−91.2618
Upper Iowa	Upper Iowa River near Dorchester, IA	10030001	05388250	291	291	1,994	43.42108	−91.5088
Wapsipinicon	Wapsipinicon River near De Witt, IA	10820001	05422000	431	428	6,050	41.76697	−90.5349
Yellow	Yellow River near Ion, IA	10030002	05389000	381	380	546	43.11193	−91.2651

### 2.2 Evaluating surrogate suitability

As a first step in evaluating surrogates for P, we explored the relationship between OP and Part P. While TP and OP concentrations are measured, Part P is not measured directly and was defined for each sample by subtracting the OP concentration from the TP concentration. This definition of PartP is not strictly accurate, as it includes dissolved organic forms of P and slightly overestimates true PartP concentrations. Still, it was necessitated because OP is the only dissolved P form with widespread historical monitoring in Iowa. OP has consistently comprised 90–95 % of total dissolved P in the few instances where it has been measured. All P concentrations reported herein use units of mg/L as P.

**Fig. 2:**
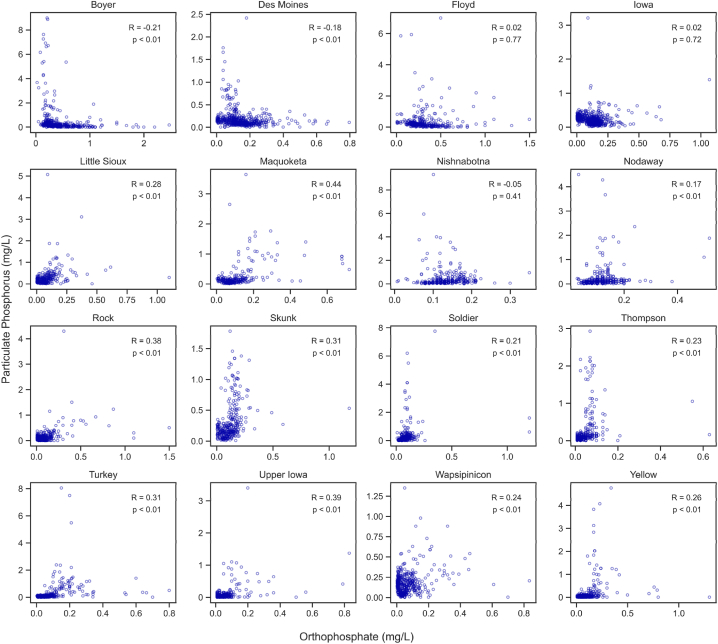
Relation between Part P and OP at the 16 terminal monitoring sites.

In rare cases, OP concentrations were greater than those of TP. As this is a physical impossibility and occurs as a laboratory artifact only when P concentrations are very low, these data were considered to be 0.00 mg/L. Table S2 lists the number of such occurrences per site. OP measurements were occasionally censored, with concentrations below a detection limit (approximately 10 % of samples). The detection limit for P forms has been 0.01 mg/L for the past 20 years. Earlier samples had higher (0.05 mg/L or 0.1 mg/L) detection limits due to less precise analytical techniques. When non-detects occurred, OP values were set to half this detection limit. Setting the non-detects to half their limit provides a simple way to fully include these data without considerable effort in adjusting them. This method has been implemented successfully in related studies [33,60]. Scatterplots were created to examine the Part P vs. OP relationship visually, and correlations were calculated (Fig. 2).

Eight potential surrogates were considered in our analysis: Chl a, DO, nitrate, pH, SC, Temp, turbidity, and flow. To be a viable surrogate, an analyte must have the potential to be measured at a higher frequency in a river at a relatively low cost [25,26]. Each of the surrogates gathered here fits this description and has been used for surrogacy purposes in the past in one way or another [20,30,45,[61], [62], [63]]. They were also all measured alongside P in the IDNR and USGS collected samples. Additional details describing the surrogates, including units and analytical methods, are provided in the supplemental materials.

Simple linear regression was constructed to evaluate the individual significance of each potential predictor with the three forms of P (TP, OP, and Part P). This method used the forms of P as separate dependent variables—evaluating their distinct response to each surrogate. Several models were created at each terminal site. Models used ordinary least squares to fit a simple linear regression model that utilized a surrogate to predict P concentrations. The models had the general form:

(1) $$\begin{array}{c} P_{predicted}=\beta_{0}+\beta_{1}*X \end{array}$$

where P_predicted_ is the estimated value of the dependent variable (P concentration), β_0_ is the intercept, β_1_ is the slope coefficient, and X is the independent variable (potential surrogate). All model estimation was conducted using the python statsmodel package (OLS linear model).

Separate models were constructed for all eight surrogates that estimated TP. This process was repeated using Part P and then OP as the dependent variables. The t-statistic corresponding to the slope coefficient was calculated in each model iteration to determine the surrogate’s statistical significance.

### 2.3 Final model construction

We further explored the specific relationship between Part P and turbidity to create final turbidity-based models that could be used for Iowa rivers. Although the relationship between turbidity and Part P can vary among rivers, we focused on creating a single model format that could be deployed across all 16 sites [20]. A single model structure provides for better interpretability [64] and helps researchers communicate results to the public [9].

Since simple linear models have issues addressing heteroskedasticity and autocorrelation among the residuals, we used a power regression model. Power regression is equivalent to taking the natural logarithm transformation of both the predictor and response variables and then performing linear regression. In addition to linear models, many surrogacy approaches have employed power regression models successfully [30,40,42,65]. A power regression model followed the general format:

(2) $$\begin{array}{c} \ln\left[Part\;P_{predicted}\right]=\beta_{0}+\beta_{1}*\ln\left[Turb\right] \end{array}$$

where Part P_predicted_ is the predicted Part P concentration, β_0_ is the intercept, β_1_ is the slope coefficient, and Turb is the measured turbidity observation. Since values of 0 are undefined for logarithmic transformations, all 0.0 mg/L Part P samples were set to 0.005 mg/L (half the typical OP detection limit) before transformation. Various metrics, such as the coefficient of determination (R^2^) and root mean square error (RMSE), were calculated for each model. The power regression models were retransformed using exponentiation to convert the model coefficients back to their original units. The retransformation process often makes the model easier to interpret. In our analysis, it takes the general form:

(3) $$\begin{array}{c} Part\;P_{predicted}=\exp\left(\beta_{0}\right)*{Turb}^{\beta_{1}} \end{array}$$

where β_0_ and β_1_ coefficients from the log-transformed linear model.

## 3 Results

### 3.1 Relationship between P species

Part P concentrations at the terminal sites ranged between 0.0 and 8.0 mg/L. In contrast, OP concentrations were considerably lower, ranging between 0.0 and 2.2 mg/L. Both datasets demonstrated positive skewness at all locations. Part P and OP samples routinely had low concentrations, but on occasion, P concentrations were orders of magnitude higher.

No strong correlations were found between Part P and OP at any site (Fig. 2, mean 0.17 ± 0.19). Many of the correlation values were close to 0 and, in some cases, were negative. The Boyer, Des Moines, and Nishnabotna sites all had negative correlations. The greatest positive correlations were 0.41, 0.40, and 0.38 at the Maquoketa, Rock, and Upper Iowa rivers, respectively. While several correlations between Part P and OP were statistically significant (p < 0.05), a higher value in one parameter was not necessarily associated with a higher value in the other. Therefore, it is difficult to infer the concentration of riverine Part P if only the OP concentration is known, as these constituents appear unrelated.

### 3.2 Surrogate suitability

**Fig. 3:**
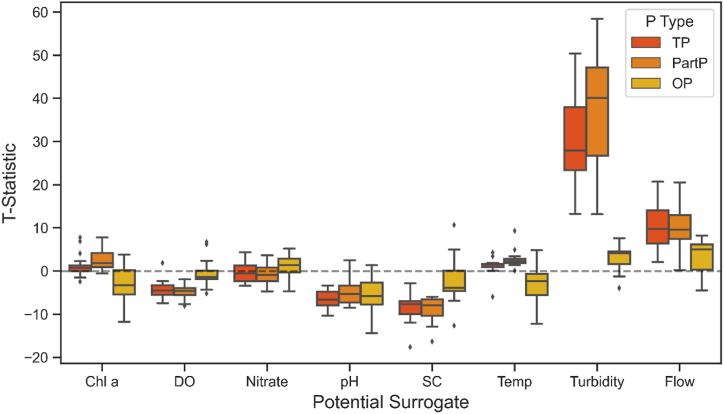
Boxplot of linear model T-statistics. These models investigate the statistical significance between P species and potential surrogates at the 16 terminal monitoring sites.

Boxplots of the t-statistics for each linear model are contained in Fig. 3. This figure summarizes the statistical significance between TP, Part P, and OP and the potential surrogates across Iowa. The supplemental materials list every model’s individual t-statistic, sample count, and R^2^ value. Turbidity was by far the most significant predictor evaluated. For TP and Part P, it was statistically significant in each river (p ≪ 0.05). Notably, the turbidity t-statistics were always larger for Part P (median of 39.5) compared to TP (median of 26.2), suggesting turbidity is more indicative of Part P than TP. By contrast, turbidity t-statistics for OP were low (median of 4.6) and indicated a weak relationship between these parameters. While turbidity’s t-statistics for PartP were always large, they varied tremendously (minimum of 12.0, maximum of 58.5), indicating the strength of the relationship between PartP and turbidity is quite variable across the sites.

Results for the rest of the surrogates were more mixed. No surrogate was found to be statistically significant (p < 0.05) at all sites. Although flow had some larger t-statistics for TP and Part P (median of 9.8 and 9.7), they were never as large as they were for turbidity. DO, pH, and SC mostly had negative t-statistics for TP and Part P, albeit much lower in magnitude than those for turbidity.

The OP models performed poorly compared to Part P. Although several surrogates were statistically significant in occasional cases, there was not a universal relationship that could be applied to all sites in a consistent manner. In several cases, the OP results had a different magnitude than those of Part P. Chl a and Temp were negative for OP but largely positive for Part P.

### 3.3 Summary of final models

**Fig. 4:**
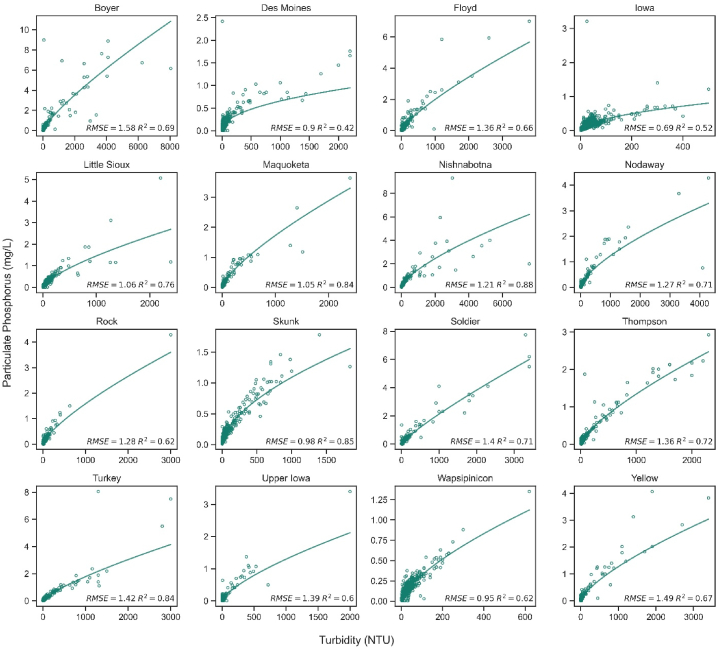
Final power regression models using turbidity as a surrogate for Part P.

Final power regression models using turbidity to predict Part P were created at all 16 terminal sites (Fig. 4). The models performed well; the mean R^2^ and RMSE values were 0.69 ± 0.12 and 1.21 ± 0.24, respectively. The slope coefficients (β1) were consistently less than 1, resulting in curvilinear models where Part P concentrations level off at higher turbidities. The model coefficients and retransformed equations are listed in Table 2. Using the power regression models seemed to alleviate the previous concerns with simple linear regression. The models now have minimal heteroskedasticity, and the residuals are more normally distributed.

**Table 2:** Results of the turbidity-based surrogacy models.

site	R^2^	RMSE	β_0_	β_1_	Retransformed Equation
Boyer	0.69	1.58	−4.888	0.8085	PartP = 0.0075 × Turb ^ 0.8085
Des Moines	0.42	0.90	−3.637	0.4657	PartP = 0.0263 × Turb ^ 0.4657
Floyd	0.66	1.36	−4.724	0.7836	PartP = 0.0089 × Turb ^ 0.7836
Iowa	0.52	0.69	−3.493	0.5281	PartP = 0.0304 × Turb ^ 0.5281
Little Sioux	0.76	1.06	−4.482	0.7034	PartP = 0.0113 × Turb ^ 0.7034
Maquoketa	0.84	1.05	−4.550	0.7380	PartP = 0.0106 × Turb ^ 0.738
Nishnabotna	0.88	1.21	−4.409	0.6978	PartP = 0.0122 × Turb ^ 0.6978
Nodaway	0.71	1.27	−4.411	0.6695	PartP = 0.0121 × Turb ^ 0.6695
Rock	0.62	1.28	−4.722	0.7499	PartP = 0.0089 × Turb ^ 0.7499
Skunk	0.85	0.98	−3.948	0.5838	PartP = 0.0193 × Turb ^ 0.5838
Soldier	0.71	1.40	−5.116	0.8494	PartP = 0.006 × Turb ^ 0.8494
Thompson	0.72	1.36	−4.783	0.7350	PartP = 0.0084 × Turb ^ 0.735
Turkey	0.84	1.42	−4.731	0.7685	PartP = 0.0088 × Turb ^ 0.7685
Upper Iowa	0.60	1.39	−4.628	0.7076	PartP = 0.0098 × Turb ^ 0.7076
Wapsipinicon	0.62	0.95	−4.284	0.6841	PartP = 0.0138 × Turb ^ 0.6841
Yellow	0.67	1.49	−4.674	0.7116	PartP = 0.0093 × Turb ^ 0.7116

A multiple linear regression approach was also explored, but supplementing Part P-turbidity models with additional predictor variables did not result in meaningful improvements. Model performance was not appreciably better than when other surrogates were included with turbidity. The differences between R^2^ (for the turbidity-only models) and adjusted R^2^ (for the multiple linear models) were slight—0.01 or less for most of the rivers. Full results of the multiple linear regression modeling have been included in the supplemental materials.

## 4 Discussion

### 4.1 Non-relationship between part P and OP

Our analysis demonstrated that Part P and OP are largely transported independently. Turbidity is commonly indicative of suspended sediment in rivers [[65], [66], [67]], and it only captures the contributions of Part P. In contrast, OP is a dissolved constituent, and dissolved constituents have a much more minor, and often insignificant, influence on turbidity [68]. This is reflected in our t-statistics, which were consistently higher for the Part P models than their TP counterparts and often insignificant for OP. In the monitoring data, numerous samples contained high concentrations of Part P and low values of OP, and vice versa, and their concentration ratio constantly varies among samples and watersheds. This general pattern of positively skewed P concentrations is aligned with other study’s findings on P dynamics [19,69]. Like many waterborne parameters, P exhibits skewed behavior that is often lognormally distributed [[70], [71], [72]]. The variety of P sources and transport pathways in Iowa can lead to impairments stemming from Part P, OP, or both simultaneously [13,73].

Previous studies using turbidity to predict TP have introduced error by including the OP as part of the model’s dependent variable (e.g. Ref. [20]). Since turbidity is only indicative of the particulate forms of P and not the dissolved sources, reasonably good fits of turbidity-based models to predict TP are due to the fact that Part P is the dominant form at large TP concentrations. This phenomenon results in co-linearity between Part P and TP, and it is this co-linearity that has resulted in the good performance of earlier models that estimate TP. In these models, Part P is the true dependent variable estimated by turbidity, while OP is added statistical noise. Therefore, a modeling approach is needed that estimates Part P and OP separately. TP concentrations can be estimated by combining the estimates from these separate models.

### 4.2 Turbidity is the only surrogate needed for part P

Turbidity proved to be a more useful surrogate than the seven others evaluated. The t-statistics describing the relationship between Part P and turbidity were far greater than those of any other models, and models that incorporated other surrogates alongside turbidity yielded only marginal improvements. These results point towards turbidity as the only predictor needed in estimating Part P in Iowa rivers. Additionally, considerable effort and financial resources are required in the collection of other surrogate data. Models that rely on numerous surrogates are not only more expensive to maintain but also more prone to implementation disruptions [74]. These models have a greater chance of not working, as fouling of a single sensor can negate the model’s ability to predict Part P. This added operational complexity further suggests deploying a turbidity-only model. Principally, at the statewide scale, the single-predictor power regression model has implementation advantages over more complex nonparametric models that require several predictors.

It was also notable that the parameters and performance of these turbidity-based power regression models varied considerably (Table 2). These variations are commensurate with the site-specific nature of surrogacy models noted by other researchers [20,24]. The 16 sites analyzed in this study contain a wide range of upstream soil and landscape characteristics [57]. P soil content and transport have been observed to vary drastically throughout the state [21,75]. Certain rivers also contain hydraulic impoundments (e.g., reservoirs or low-head dams) that impact P transport. These differences in upstream geologic and hydrologic conditions are responsible for the variation noted among the 16 models, but the exact influence of upstream conditions on model parameters is not fully understood. Future analyses could investigate linking spatial characteristics to surrogacy model parameters for P.

### 4.3 Nonlinear relationship between part P and turbidity

An important result of the final model construction was the nonlinear nature of the relationship between turbidity and Part P. For all 16 terminal sites, a power regression model fit the data better than a model created from simple linear regression. Similar behavior has been noted in other studies [40,56]. However, we questioned whether the curvilinear model was strictly a function of turbidity measurement or representative of a broader environmental process.

Total suspended solids (TSS) concentration is a measure of the entire body of particulate matter present within a water column. Turbidity is typically considered an indirect indicator of TSS [76]. Part P is included within TSS, generally as a tiny percentage [77,78]. We assembled the TSS data collected concurrently with P at the 16 sites to determine if the relationships between TSS and turbidity differed from those observed in the turbidity-Part P models. It should be noted that TSS itself is not a viable surrogacy option for predicting Part P. It is measured by completely drying a sample under specific, controlled conditions and then weighing its remaining matter [65]. This procedure requires laboratory accommodations and cannot be performed in the field continuously [68].

**Fig. 5:**
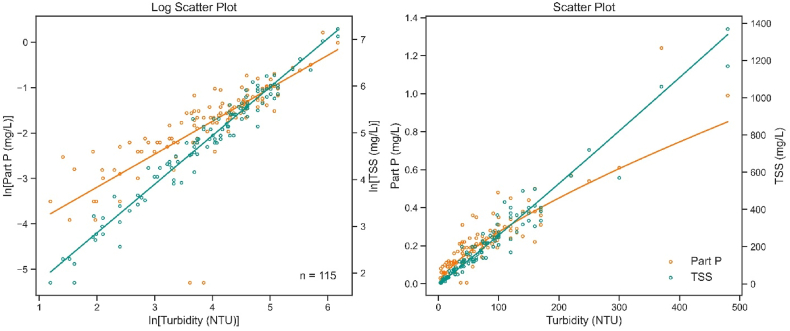
Power regression of results of Part P vs. turbidity and TSS vs. turbidity at the Little Sioux site. Left: log-transformed simple linear regression. Right: retransformed regression models.

Differences were evident when both Part P and TSS were plotted against turbidity, and two power regression models were fit (Fig. 5). Whereas the Part P model retained its nonlinear nature (slope of 0.67), the TSS model was highly linear (slope of 1.02). The slopes indicate that the overall amount of particulate matter varies proportionately to turbidity, but Part P does not. The proportion of Part P wanes as more sediment gets deposited. The analysis was conducted at each terminal site, and the same behavior was observed. Table S1 lists all the models’ slopes. The Part P slopes had a mean of 0.68 ± 0.14, whereas the TSS slopes had a mean of 1.02 ± 0.05. It was especially noteworthy that the slopes for all Part P models were less than 1, while the slopes for all TSS models were extremely close to 1. TSS and turbidity are strongly linear, while the relationship between Part P and turbidity is curved and increases more slowly than a linear one. This range in parameters among sites indicates that the relationship between Part P and turbidity is not universal. Instead, it is based on environmental and regional conditions and may even be site-specific.

The results of this analysis suggest power regression models should be used when dealing with turbidity and Part P. As seen across Iowa, Part P consistently increases when sediment increases in a river, but the increase becomes more gradual as larger amounts of sediment are eroded. Different nonpoint sources of P may be more present during periods of high turbidity, resulting in the nonlinear relationship between Part P and turbidity. Increased turbidity is often the result of wet weather, which triggers a spike in erosion [79]. Under these conditions, the sediment making its way to the stream may have a lesser P makeup than what is observed in the soils present during more typical flows. Bedload P may also be a factor, as high flow in Iowa rivers results in greater bedload transport [80]. Concentrations of P are often smaller in bedload sediment than in newly eroded soils [81], which likely explains some of the waning Part P concentrations at higher turbidities.

### 4.4 Lack of surrogates for OP

No surrogates proved viable for routinely estimating OP; there was no equivalent to the strong relationship between turbidity and Part P. It is important to quantify OP because it is a harmful pollutant and a nontrivial component of Iowa’s TP loads. OP is the primary form of P bioavailable to plants [82,83] and presents the most immediate risks associated with eutrophication [84].

The non-relationship between OP and potential surrogates is interesting for various reasons. Chl a is a compound used to gauge the amount of algal growth in a waterbody [85,86]. Since OP is most often the limiting nutrient for algal blooms in Iowa [3], it stands to reason that it would be correlated with Chl a, but no such relationship was found. Instead, OP was inversely related to Chl a in some rivers, and it may be the case that in such instances, OP was consumed by algal organisms, depleting its concentration in the water column. The processes that drive algal blooms are complex, chaotic, and not fully understood [87]. Elevated levels of OP are required for algal blooms to occur, but many other factors control algal growth [88]. It is likely that when high OP levels occur in Iowa’s rivers, other conditions may be inhibiting algal growth.

SC has been implemented in several studies as a surrogate for dissolved constituents [[89], [90], [91]], most notably total dissolved solids [92]. However, it was not related to OP. In Iowa waters, OP makes up a tiny percentage (<1 %) of total dissolved solids, and the processes that drive OP are likely unrelated to those that drive dissolved constituents as a whole.

Nitrate, a common dissolved form of nitrogen, also plagues Iowa water resources [11,93]. Nitrate is mainly delivered to Iowa streams through groundwater and tile flow [94]. Streamflow is often linked to pollutants delivered through surface water runoff [95]. Large streamflow values in Iowa indicate significant overland flow has occurred through the agricultural or urban landscapes [96]. However, both nitrate and streamflow were unrelated to OP. These findings suggest riverine OP may have a pathway not directly linked to events that trigger large overland or subsurface nutrient movement, and the supply of OP on the landscape may be more easily depleted than the supply of nitrate. The exact mechanisms contributing OP to Iowa’s waterbodies are a mixture of runoff, subsurface, and seasonal processes [97], but their exact components are not fully understood [19]. Reliably estimating OP in Iowa waterbodies remains elusive, and future analyses could explore utilizing more sophisticated modeling techniques to predict its presence.

### 4.5 Implementation considerations

The power regression models utilizing turbidity have great potential in estimating Part P across Iowa, generating real-time concentrations, and quantifying annual loads. The approach proved viable in estimating Part P at the statewide scale. Our approach could also be duplicated in other areas of the world with similar repositories of water quality and streamflow data to develop new surrogacy models. However, there are occasional challenges associated with collecting riverine turbidity over long periods. Turbidimeters must be deployed at all 16 terminal sites to estimate statewide Part P concentrations. In a Midwestern state such as Iowa, turbidity devices must be retrieved during freezing conditions [98], and some sensor fouling issues are inevitable [30]. Future work could explore additional modeling strategies (e.g., machine learning techniques), but implementing more advanced models is likely to incur a greater number of challenges than the ones developed in this study.

To address periods when turbidity sensors are not active, we suggest using other statistical methods to supplement the turbidity-based models for Part P. The USGS has developed statistical packages to evaluate daily constituents when only discrete samples are available, including models such as WRTDS [99] and LOADEST [100]. These models can be created solely using existing IDNR and USGS data and do not require additional in-situ monitoring. These statistical models for Part P can act as a backup model on days when turbidity data are unavailable. The WRTDS model is the most effective method for modeling Iowa’s OP [101]. In combination, the turbidity-based models and statistical models can be used to create accurate estimates of Iowa’s P behavior and track progress of the INRS.

## 5 Conclusions

This study investigated the relationship between eight potential surrogates and various forms of P at Iowa’s 16 terminal monitoring sites. Data were gathered from the IDNR’s ambient monitoring program and the USGS’s Big River Study, which have generated robust water quality datasets from 1998 to present. Study results showed that the correlation between OP and Part P was poor and necessitated the development of separate models, one for Part P and one for OP. Simple linear models were used to explore the statistical significance among the eight surrogates and P forms. For Part P, turbidity-based surrogacy models were the most significant in all cases, and adding additional surrogates to turbidity alone did not improve model performance. For the OP models, none of the surrogates proved viable, with no single predictor being consistently significant. OP models generally did not perform well, indicating that OP surrogates were not viable. A power law regression best described the relation of Part P to turbidity for all rivers. The surrogacy approach to estimate Part P enables real-time and high-resolution estimation of P concentrations that will be needed to help track progress toward P reduction efforts in Iowa.

## Data availability statement

All water quality data used in this study can be retrieved through the EPA STORET database (https://www.epa.gov/waterdata/water-quality-data) or the IDNR AQuIA database (https://programs.iowadnr.gov/aquia/). All streamflow data used in this study can be retrieved through the USGS National Water Information System (https://waterdata.usgs.gov/nwis).

## CRediT authorship contribution statement

**Elliot S. Anderson:** Writing – review & editing, Writing – original draft, Visualization, Methodology, Data curation, Conceptualization. **Keith E. Schilling:** Writing – review & editing, Visualization, Supervision, Methodology, Funding acquisition, Conceptualization. **Chris S. Jones:** Writing – review & editing, Supervision, Methodology, Conceptualization. **Larry J. Weber:** Supervision, Project administration, Methodology, Funding acquisition, Conceptualization.

## Declaration of competing interest

The authors declare the following financial interests/personal relationships which may be considered as potential competing interests:Elliot Anderson reports financial support was provided by The 10.13039/100011460Iowa Department of Natural Resources.
