# Supplementary material for: Estimating Iowa’s riverine phosphorus concentrations via water quality surrogacy
Source: Heliyon. 2024 Sep 3;10(17):e37377. doi: 10.1016/j.heliyon.2024.e37377 (PMC11408025; doi:10.1016/j.heliyon.2024.e37377)
Supplement: Multimedia component 4 [file mmc4.docx]

# Supplemental Materials

Table S1. Slope parameters from power regression models. (Part P vs. Turbidity; TSS vs. Turbidity)

| **Power Regression Slopes** | | |
| --- | --- | --- |
| **River** | **Part P** | **TSS** |
| Boyer | 0.9226 | 1.0578 |
| Des Moines | 0.3569 | 0.9387 |
| Floyd | 0.8059 | 1.0147 |
| Iowa | 0.5 | 1.0305 |
| Little Sioux | 0.7266 | 1.0399 |
| Maquoketa | 0.7069 | 0.9747 |
| Nishnabotna | 0.7476 | 1.0158 |
| Nodaway | 0.6727 | 1.0235 |
| Rock | 0.7098 | 1.0298 |
| Skunk | 0.5393 | 1.0673 |
| Soldier | 0.8458 | 1.0138 |
| Thompson | 0.7329 | 1.1225 |
| Turkey | 0.722 | 0.97 |
| Upper Iowa | 0.6077 | 1.0094 |
| Wapsipinicon | 0.6957 | 1.118 |
| Yellow | 0.6031 | 1.0361 |
| *mean* | *0.681* | *1.029* |
| *median* | *0.708* | *1.027* |
| *stdev* | *0.137* | *0.048* |

Table S2. P samples counts per site. The final column lists the number of samples where measured OP concentrations where greater than TP. PartP concentrations in these samples were set to 0.00 mg/L.

| **Site** | **TP Samples** | **OP Samples** | **PartP Samples** | **Samples where OP>TP** |
| --- | --- | --- | --- | --- |
| Boyer | 397 | 399 | 394 | 23 |
| Des Moines | 509 | 510 | 509 | 4 |
| Floyd | 306 | 306 | 303 | 9 |
| Iowa | 496 | 497 | 495 | 0 |
| Little Sioux | 254 | 254 | 254 | 2 |
| Maquoketa | 254 | 254 | 254 | 0 |
| Nishnabotna | 255 | 255 | 255 | 2 |
| Nodaway | 274 | 273 | 271 | 7 |
| Rock | 276 | 273 | 273 | 6 |
| Skunk | 288 | 289 | 288 | 0 |
| Soldier | 284 | 284 | 281 | 4 |
| Thompson | 280 | 278 | 275 | 0 |
| Turkey | 449 | 415 | 415 | 3 |
| Upper Iowa | 291 | 291 | 288 | 11 |
| Wapsipinicon | 431 | 428 | 428 | 3 |
| Yellow | 381 | 380 | 377 | 15 |
| *ALL SITES* | *5425* | *5386* | *5360* | *89* |

**Chemical Forms of Phosphorus Summary**

- TP: Total Phosphorus (as P) in mg/L
  - Analysis Method: LACHAT Method LAC 10-115-01-1-F
  - Note: This parameter contains all forms of phosphorus present in a water sample.
- OP: Orthophosphate (as P) in mg/L
  - Analysis Method: LACHAT Method LAC 10-115-01-1-A
  - Note: Orthophosphate is the simplest and most common form of phosphate. It is the most prevalent dissolved form of P in Iowa’s surface waters and consistently makes up ~90% of dissolved P concentrations.
- Part P: Particulate Phosphorus (as P) in mg/L
  - Analysis Method: not measured directly
  - Note: Part P was inferred in sample by subtracting OP from TP. In rare instances when OP was greater than TP (a physical impossibility), Part P was set to 0.0 mg/L.

**Potential Surrogate Summary**

- Chl a: Chlorophyll a in µg/L
  - Analysis Method: EPA Method 445.0
  - Note: Chlorophyll a has been corrected, so the pheophytin is not included in the concentration. This is the type of chlorophyll that is indicative of phytoplankton.
- DO: Dissolved Oxygen in mg/L
  - Analysis Method: ASTM Method D888(C)
- Nitrate: Nitrate plus Nitrite (as N) in mg/L
  - Analysis Method: LACHAT Method LAC 10-107-04-1-J
  - Note: This is sometimes referred to as inorganic nitrogen. Nitrite is very unstable in aerobic aquatic environments, so nitrate makes up the vast majority of nitrogen in these samples.
- pH: standard units
  - Analysis Method: ASTM Method 4500-H
- SC: Specific Conductivity in µS/cm
  - Analysis Method: ASTM Method 2510
  - Note: The conductivity of the water is adjusted to its equivalent conductivity at 25 °C. The ratio between SC and total dissolved solids (TDS) is known to be relatively constant at a specific site. This ratio was calculated for each site from observations that measured both SC and TDS. Some observations only measured TDS; in these cases, SC was quantified using the specific ratio for each site with the TDS measurement.
- Temp: water Temperature in °C
  - Analysis Method: ASTM Method 2550
- Turbidity: water Turbidity; units for IDNR are NTU, units for USGS are varied but mainly in NTU
  - Analysis Method: ASTM Method 2130-B
  - Note: IDNR measurements consistently used NTU, and the USGS used NTU for about 80% of their measurements. They employed a few additional turbidity units for the remainder, usually FNU.
- Flow: Streamflow in cfs
  - Analysis Method: USGS standard protocol
  - Note: Daily mean flow values based on 15-minute measurements taken by the USGS.
